# Leicester as a Hospital Town

**Published:** 1915-02-20

**Authors:** 


### LEICESTER AS A HOSPITAL TOWN.

### The Problem of Extended Accommodation.

By A CORRESPONDENT.

Inquiries have been made during the past week by the War Office authorities as to the possibility of accommodating 2,000 additional sick and wounded in Leicester. With this object in view, a deputation has inspected several of the large schools in the borough which, it is understood, were found suitable for use as temporary hospital buildings. The De Montford Hall, newly erected by the Corporation for musical and other gatherings, was also visited and found adaptable for hospital purposes. Including the various private dwellings and buildings which have already been equipped as supplemental hospitals, the 5th Northern General Hospital controls at present about 1,000 beds, so that it is apparently the intention of the War Office to call upon the town and county to provide for 3,000 sick and wounded. The reasons given for this increased hospital accommodation in Leicester are the central situation of the town, the ample railway and transport facilities, a good supply of doctors, and a desire to retain the coast hospitals for emergencies.

The proposal is one which is not without many difficulties. The medical men of the town and county since the outbreak of war have had their numbers considerably diminished by mobilisation, and this has imposed additional work upon the medical men remaining, who have voluntarily and readily undertaken the work of their colleagues so mobilised. The medical staff of the 5th Northern General Hospital is apparently well-nigh fully occupied in dealing with the medical and surgical treatment of patients in that institution, and there is, of course, a limit to the additional number of patients which they can take the responsibility for. The supply of trained nurses, whether for public or private purposes, is certainly very limited, so that it is essential that the authorities realise that they will have largely to rely for the nursing of further cases on nurses belonging to Voluntary Aid Detachments, and certainly there is a limit to the work which the St. John Ambulance members are able to do voluntarily in connection with the transport of patients, and possibly to the number of motor-cars at the disposal of the County Director.

From an administrative point of view, the suggestion to equip buildings in various parts of the town is accompanied with difficulties, for it is quite evident that medical, nursing, and commissariat supervision cannot be so satisfactory in decentralised units. Many alterations would have to be made in the structure, heating and lighting, and equipment of the buildings, and sleeping accommodation for doctors and nurses would require much careful thought. It is possible, however, that it is the intention of the authorities to equip the buildings for convalescent soldiers and not for those needing active surgical and medical treatment, but the transformation of schools into convalescent hospitals cannot be undertaken without great expense in the remodelling of the sanitary arrangements, the supply of bathing accommodation, and the provision of adequate culinary departments—in fact most of the amenities which are indispensable in a modern hospital.

The education authorities of the town would necessarily have to reorganise their system to provide for the training of the scholars who would be displaced by the acquisition of school buildings for military purposes. This must be mentioned, since it has to be borne in mind that there is no supplemental accommodation available.

These points are enumerated for the purpose of suggesting to the authorities, if they really contemplate the increase of the hospital accommodation of Leicester, that it would be wise to take steps to organise a reserve staff of officers, doctors, nurses, and orderlies, under the control of the Commandant of the 5th Northern General Hospital, for immediate training and for mobilisation as and when required—only by such a step, it seems, could the treatment of a large additional number of wounded and sick soldiers be undertaken. The alternative is presumably an immediate enlargement of the base hospital and the consequent augmentation of the entire staff of that institution—a course which would have much to commend it as a more satisfactory solution of the problem now presented, provided, of course, that new temporary buildings could be erected within a stipulated time. The problem presented is an interesting one, and the measures adopted for the accommodation, medical care, and nursing of this large additional number of patients will be awaited with interest.

